# Supplementary material for: A Comprehensive Assessment of Ultraviolet-Radiation-Induced Mutations in Flammulina filiformis Using Whole-Genome Resequencing
Source: J Fungi (Basel). 2024 Mar 20;10(3):228. doi: 10.3390/jof10030228 (PMC10971301; doi:10.3390/jof10030228)
Supplement: Supplementary file 1 [file jof-10-00228-s001.zip › Supplementary Material S8/KEGG annotation/out/64381550635650.os/KO/out_map/map03030.html]

KEGG PATHWAY: DNA replication - Reference pathway


|  |  |
| --- | --- |
| **DNA replication - Reference pathway** |  |

[
Pathway menu
| Organism menu
| Pathway entry
| Show description
| User data mapping
]

A complex network of interacting proteins and enzymes is required for DNA replication. Generally, DNA replication follows a multistep enzymatic pathway. At the DNA replication fork, a DNA helicase (DnaB or MCM complex) precedes the DNA synthetic machinery and unwinds the duplex parental DNA in cooperation with the SSB or RPA. On the leading strand, replication occurs continuously in a 5 to 3 direction, whereas on the lagging strand, DNA replication occurs discontinuously by synthesis and joining of short Okazaki fragments. In prokaryotes, the leading strand replication apparatus consists of a DNA polymerase (pol III core), a sliding clamp (beta), and a clamp loader (gamma delta complex). The DNA primase (DnaG) is needed to form RNA primers. Normally, during replication of the lagging-strand DNA template, an RNA primer is removed either by an RNase H or by the 5 to 3 exonuclease activity of DNA pol I, and the DNA ligase joins the Okazaki fragments. In eukaryotes, three DNA polymerases (alpha, delta, and epsilon) have been identified. DNA primase forms a permanent complex with DNA polymerase alpha. PCNA and RFC function as a clamp and a clamp loader. FEN 1 and RNase H1 remove the RNA from the Okazaki fragments and DNA ligase I joins the DNA.

|  |  |
| --- | --- |
| Reference pathway | 100% |
